# Deep learning-based anatomical site classification for upper gastrointestinal endoscopy

**DOI:** 10.1007/s11548-020-02148-5

**Published:** 2020-05-06

**Authors:** Qi He, Sophia Bano, Omer F. Ahmad, Bo Yang, Xin Chen, Pietro Valdastri, Laurence B. Lovat, Danail Stoyanov, Siyang Zuo

**Affiliations:** 1 Key Laboratory of Mechanism Theory and Equipment Design of Ministry of Education, Tianjin University, Tianjin, China; 2 Wellcome/EPSRC Centre for Interventional and Surgical Sciences (WEISS), University College London, London, UK; 3 General Hospital, Tianjin Medical University, Tianjin, China; 4 School of Electronic and Electrical Engineering, University of Leeds, Leeds, UK

**Keywords:** Artificial intelligence, Endoscopy, Gastroenterology, Deep learning

## Abstract

**Purpose:**

Upper gastrointestinal (GI) endoscopic image documentation has provided an efficient, low-cost solution to address quality control for endoscopic reporting. The problem is, however, challenging for computer-assisted techniques, because different sites have similar appearances. Additionally, across different patients, site appearance variation may be large and inconsistent. Therefore, according to the British and modified Japanese guidelines, we propose a set of oesophagogastroduodenoscopy (EGD) images to be routinely captured and evaluate its efficiency for deep learning-based classification methods.

**Methods:**

A novel EGD image dataset standardising upper GI endoscopy to several steps is established following landmarks proposed in guidelines and annotated by an expert clinician. To demonstrate the discrimination of proposed landmarks that enable the generation of an automated endoscopic report, we train several deep learning-based classification models utilising the well-annotated images.

**Results:**

We report results for a clinical dataset composed of 211 patients (comprising a total of 3704 EGD images) acquired during routine upper GI endoscopic examinations. We find close agreement between predicted labels using our method and the ground truth labelled by human experts. We observe the limitation of current static image classification scheme for EGD image classification.

**Conclusion:**

Our study presents a framework for developing automated EGD reports using deep learning. We demonstrate that our method is feasible to address EGD image classification and can lead towards improved performance and additionally qualitatively demonstrate its performance on our dataset.

## Introduction

Oesophagogastroduodenoscopy (EGD) is the gold-standard investigative procedure in the diagnosis of upper gastrointestinal (GI) diseases, such as reflux oesophagitis, gastroduodenal ulcer and particularly for the detection of early gastric cancer [13]. EGD is widely performed especially in geographical regions with high disease incidence. Early gastric cancer and other significant pathology can be easily missed during EGD due to potential blind spots. A preliminary study suggested that longer examination times and more captured pictures may improve the detection of lesion [22]. Therefore, mapping of the upper GI tract through the use of standardised photo-documentation is considered a quality indicator. Using artificial intelligence (AI) to understand the endoscopic examination process can potentially help EGD clinicians to quickly quantify their photo-documentation that summarised a case and additionally could even support the detection and identification of diseased lesions [4, 17]. The use of AI for computer-assisted endoscopy can potentially support and efficiently improve the quality of endoscopy by ensuring a complete examination, by enhancing navigation with 3D mapping [2, 23] or through automated procedural analysis [24]. Deep learning-based methods are the current state-of-the-art methodology for almost all image understanding and analysis problems like semantic segmentation, image recognition and classification [6, 11]. In gastroenterology, AI methods have been explored from both the classical model-driven and deep learning paradigms [20]. While the majority of work has focused on the detection or delineation of diseased regions [5, 14, 27], on the measurement of structural size [10] or the 3D navigation within the endoluminal organs [12, 15, 25], relatively little research effort has been invested into the classification of different endoscopic viewpoints that need to be viewed to complete an examination.

Geographical regions with higher gastric disease incidence need a more complex and more complete endoscopic procedure. And as reported by the new global cancer data, GLOBOCAN 2018 estimates of cancer incidence, Eastern Asia has higher rates of stomach cancer than Western Europe [3]. As a simple instance for this, a commonly accepted Japanese (or Eastern Asian) guideline proposed in 2013, namely the systematic screening protocol for the stomach (SSS), comprises 22 endoscopic images [26], while its counterpart in Europe proposed by the British Society of Gastroenterology (BSG) and Association of Upper GI Surgeons of Great Britain and Ireland (AUGIS) in 2017 introduced only eight standard images. The Japanese SSS guideline focuses exclusively on detailed imaging of the stomach including comprehensive multiple quadrant views of each landmark. The British guideline is more pragmatic, with fewer images of the stomach but includes additional important landmarks outside of the stomach. In practice, the SSS guideline is not routinely clinically implemented outside of Japan. Therefore, we proposed a modified guideline which represents a balance between the British and Japanese standards, by merging the multiple quadrant views of the stomach. Furthermore, in this study, the dataset contained endoscopic report images from routine clinical care in China, where multiple quadrant views would not have been obtained according to the Japanese SSS protocol. These guidelines are described in more detail under “Related work” section.

The presented dataset (comprising of 3704 EGD images) is annotated by a clinical expert and a medical imaging doctoral student following the proposed guideline. This new dataset has three advantages: (a) from the clinical perspective, it can assess the quality of photo-documentation report based on British guidelines, (b) from feasibility considerations, each category within it varying from the other categories helps classifier to train and (c) from compatibility, uncertain location images or transitional images are annotated separately from other landmarks for our further video-based study. Our images are gathered from the clinical endoscopes in use at Tianjin Medical University General Hospital, Tianjin, China. Additionally, due to the variety of clinical systems in use, our images have various resolutions and regions of interest (ROI). To unify the data, we design an automatic multi-resolution ROI extraction method to extract the ROI. While simple, this method is important in our workflow. Then, all images and labels are feed to a convolutional neural network (CNN), the performance of which would show the feasibility of proposed guideline and the efficiency of proposed workflow for EGD image classification.

**Fig. 1:**
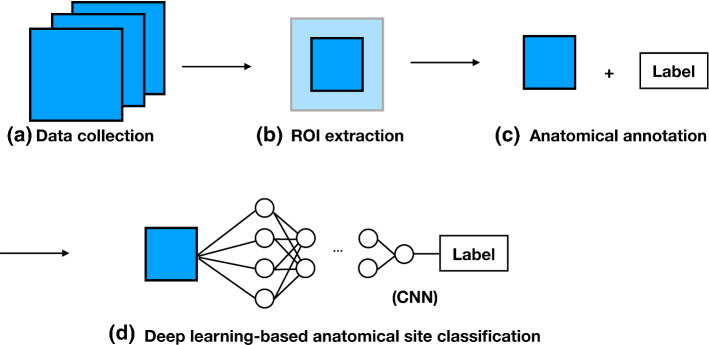
Proposed workflow

In this work, we take anatomical site localisation as a classification task consisting of four consecutive steps (Fig. 1), namely (a) data collection, (b) ROI extraction, (c) anatomical annotation and (d) deep learning-based anatomical site classification (details presented in “Methods” section). Here, we are more focussed on solving the clinical problem using previous deep learning methods because our pipeline could be easily adapted to existing classification methods. Our highlights are as follows:

- we proposed a modified guideline for upper GI endoscopic photo-documentation. This guideline is described in further “Related work” section.
- we presented a new upper GI endoscopic dataset because the annotated dataset is crucial to develop and validate the artificial intelligence system. Our dataset is intended to mitigate this gap.
- we introduced a complete workflow for EGD image classification. To the best of our knowledge, none of the existing work has described the complete workflow, including data collection and automatic ROI extraction for upper GI endoscopic image classification.

**Table 1:** Recommended images of landmarks in upper GI endoscopy

Recommendations	Oesophagus	Stomach	Duodenum
British guideline (BSG and AUGIS [1], ESGE [16])	Proximal oesophagus, Z-line	Cardia and fundus on retroflexed view, body (taken from the upper part of the less curvature), angulus on partial retroflexion, antrum	Duodenal bulb, second part of the duodenum
Japanese guideline (Yao [26])	Not defined	Four quadrants (L, G, A and P) of the fundus on retroflexed view, three quadrants (L, A and P) of middle-upper body and angulus on retroflexed view, four quadrants (L, G, A and P) of antrum, lower body and middle-upper body on forward view	Not defined
Proposed guideline (2019)	Pharynx oesophagus, gastroesophageal junction	Cardia and fundus on retroflexed view, middle-upper body on either forward and retroflexed view, lower body on forward view, angulus on retroflexed view, antrum on forward view	Duodenal bulb, duodenal descending

L, lesser curvature; G, greater curvature; A, anterior wall; P, posterior wall

## Related work

*Commonly used guidelines for endoscopic photo-documentation* Recently, upper GI endoscopic photo-documentation has gained an important role in quality assurance for endoscopic procedures (Table 1). In 2001, the European Society of Gastrointestinal Endoscopy (ESGE) published a guideline for standardised image documentation in upper GI endoscopy, recommending the acquisition of specific anatomical landmarks [16]. In 2013, Yao et al. developed its Japanese counterpart, known as SSS which involved very detailed and rigorous mapping of the entire stomach to avoid blind spots [26]. In addition to the British guideline, for each landmark in the stomach, an additional picture is required for each quadrant, for example anterior, posterior, less curvature and greater curvature. Also, mid-upper body in retroflexion is not in the British guideline. Later in 2017, the BSG and AUGIS released “Quality standards in upper GI endoscopy” [1]. For the baseline clinical examination, a minimum of eight sites needs to be identified during EGD examinations. Standardised photo-documentation guidelines are designed to reduce variation during endoscopy and serve as a surrogate marker for the quality of inspection; however, in practice endoscopists vary considerably in their ability to adhere to these guidelines. Automated computer-aided capture and classification of images according to guidelines could help overcome this.

*Automatic EGD image classification* The most recent anatomical classification methods in endoscopy are primarily based on CNNs because of the methodology’s capability to identify complex nonlinear feature spaces and features for data classification [11]. In 2018, a CNN-based method to recognise the anatomical location from 27,335 images of only six main categories (larynx; oesophagus; upper stomach; middle stomach; lower stomach; duodenum) has been reported [21]. In 2019, a deep learning- and reinforcement learning-based system named WISENSE has been proposed to monitor the blind points and time of procedure, and to classify each anatomical sites for automatically generating photo-documentation during EGD [24]. WISENSE applied the 27-class protocol (adapted from the Japanese guideline) which include 26 classes corresponding to each anatomical site and one NA class for images that cannot be classified into any site.

**Fig. 2:**
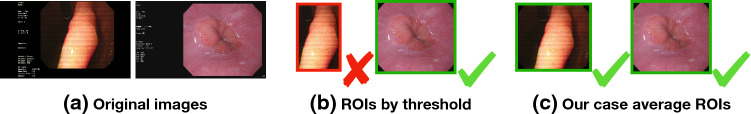
Samples of ROI extraction

## Methods

### Data collection

We acquired 5661 EGD images from a total of 229 clinical cases that include 18 endoscopic submucosal dissections (ESD) and 211 normal EGD examinations. These were acquired from Tianjin Medical University General Hospital, and the instruments used during these examinations included various gastroscopes from two vendors (Olympus Optical Co., Tokyo, Japan; Fujifilm, Co., Kanagawa, Japan). The 18 cases of ESD are excluded because of their different aims and workflows comparing to normal EGD examinations. The images from the 211 EGD cases can be divided into three categories based on their imaging type, namely (a) white light imaging (WLI) and linked colour imaging (LCI), (b) narrow band imaging (NBI), blue laser imaging (BLI) and BLI-bright (BLI-brt) and (c) chromoendoscopy. Images from WLI and the LCI are very similar in terms of tissue appearance, colour space and texture under an unmagnified imaging modality. Therefore, we use WLI and LCI images in our experiments.

The NBI and the BLI are two similar imaging modalities captured from Olympus and Fujifilm, respectively. Although these modalities allow superior visualisation of the superficial vascular and mucosal pattern under magnified modality, they are not suitable for our task as they do not provide sufficient anatomical site location information compared to other unmagnified imaging modalities. The chromoendoscopy is also excluded as their blue colour space.

The WLI and LCI images that we selected need further filtering, as it contained frames which included another small display picture, or a transparent hood which was attached to the endoscope, which resulted in unnecessary artificiality to the image. Moreover, some images contained food residue which was irrelevant for the current study as they retained less useful information. We removed all images that blocked the main display or contained food residue. The final dataset that we use in our study contained 3704 pictures of WLI and LCI images.

### ROI extraction

Cropping the colour foreground from the image by a rectangular box is a necessary pre-processing step before training model. These images need to be cropped and resized in such a way that they only contain the endoscopic camera view. A fixed global ROI setting would fail to solve the various resolutions and different endoscopic display settings because our images are captured using different instruments and imaging devices. The threshold method (Fig. 2) would also fail to extract the ROI because of the artificial texts and uneven illumination. To solve this issue, we proposed a data-driven method for cropping the colour (endoscopy view) foreground from our data. We assumed that captured pictures from the same case share the same display setting. So, instead of calculating the mask image by image, we calculate our mask case by case. Comparing to searching on the original image, searching on the case average image leads towards an improved ROI.

**Fig. 3:**
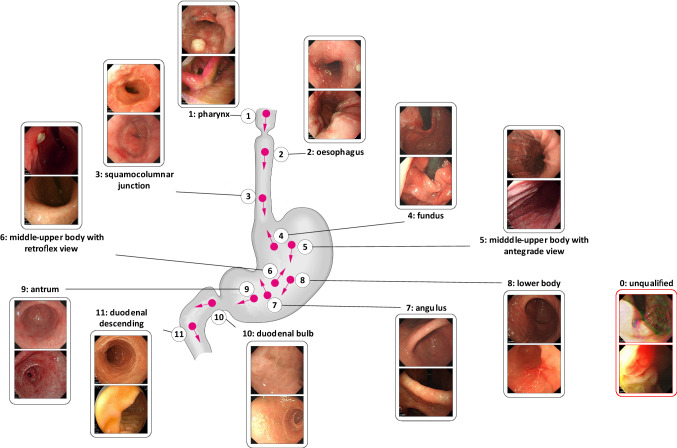
Proposed anatomical classification guideline

### Anatomical annotation and guideline

Our modified guideline represents a balance between the British and Japanese guidelines (both of which are introduced in “Related work” section). Our EGD images are co-labelled by a Chinese medical imaging doctoral student and a British clinical endoscopy research fellow based on the proposed guideline. 3704 ROI images are manually annotated into 11 landmarks or unqualified (NA) (Fig. 3). The 11 landmarks are divided into antegrade view and retroflex view. Eight landmarks with an antegrade view are pharynx (PX), oesophagus (ES), squamocolumnar junction (SJ), middle-upper body of antegrade view (MA), lower body (LB), antrum (AM), duodenal bulb (DB) and duodenal descending (DD). Three landmarks of retroflex view are fundus (FS), middle-upper body of retroflex view (MR) and angulus (AS).

### Deep learning-based anatomical site classification

CNN methods are widely used for image classification because of their strong feature representation capability. Through transfer learning, the existing pre-trained CNN models can be easily fine-tuned to incorporate the target domain. We experimented with the most widely used CNN architectures that were pre-trained on the ImageNet dataset, such as ResNet-50 [7], Inception-v3 [19], VGG-11-bn [9, 18], VGG-16-bn [9, 18] and DenseNet-121 [8] by fine-tuning these networks using our training dataset. Through transfer learning, these five CNN models for image recognition can learn anatomical site classification from our dataset. For fine-tuning, we replace the last fully connected (FC) layer of the CNN model by a fully connected layer having the same number of layers as that of the number of classes in the training set. We then apply mini-batch training and use the multi-class cross-entropy loss

1 $$\begin{aligned} L({\hat{y}},y) = -\sum _{k=1}^{K}{y^{(k)}\,\log \,{\hat{y}}^{(k)}} \end{aligned}$$

to minimise the training loss in our multi-class classification problem, where *y* is 0 or 1 if class label *k* is the correct classification, ${\hat{y}}$ is the predicted probability of class *k* and *K* is the number of classes. The losses within the same batch are accumulated during the training period of the batch and backpropagated to the previous layers at the end of each batch training to update the model weights.

## Experiments

### Materials

**Fig. 4:**
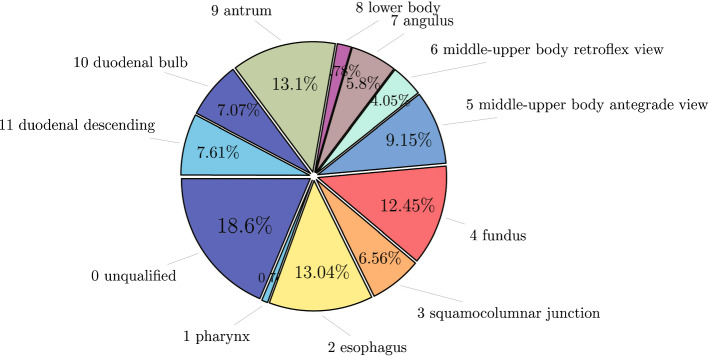
Proportion of NA and 11 anatomical landmarks

We divide the ROI data into 12 classes based on the proposed guideline. Proportions for each of the classes are shown in Fig. 4. The ROI data are arranged based on the proposed guideline and the British guideline [1, 16] and two conditions (with NA and without NA). We exclude those classes from the ROI data which are not included in the guidelines under analysis. Finally, four different forms of the dataset are generated as shown in Table 2.

To build the training and test sets from a limited number of data, we divide the dataset into five parts based on fivefold cross-validation such that each part is in the same data distribution. The CNN models are trained on threefold and validated on the other onefold and tested on the remaining onefold.

**Table 2:** Manually annotated (ground truth) labels of four training/test sets

No. (cite)	NA	PX	ES	SJ	FS	MA	MR	AS	LB	AM	DB	DD
0 (proposed)	–	0	1	2	3	4	5	6	7	8	9	10
1 (proposed)	0	1	2	3	4	5	6	7	8	9	10	11
2 ([1, 16])	–	–	0	1	2	3	–	4	–	5	6	7
3 ([1, 16])	0	–	1	2	3	4	–	5	–	6	7	8

–, does not exist; NA, unqualified; PX, pharynx; ES, oesophagus; SJ, squamocolumnar junction; FS, fundus; MA, middle-upper body antegrade view; MR, middle-upper body retroflex view; AS, angulus; LB, lower body; AM, antrum; DB, duodenal bulb; DD, duodenal descending

### Evaluation metrics and model implementation

The overall accuracy

2 $$\begin{aligned} \hbox {rate}_{oa}(Y,f(X)) = \frac{\hbox {sum}(\hbox {diag}(\hbox {CM}(Y,f(X))))}{\hbox {sum}(\hbox {CM}(Y,f(X)))}, \end{aligned}$$

is used to assess the performance of the model, where *X* is the input, *Y* is the ground truth, *f*(.) is the CNN model, $\hbox {CM}(Y, f(X))$ is the confusion matrix, $\hbox {diag}(.)$ is the diagonal of the matrix, and $\hbox {sum}(.)$ accumulates all elements in a matrix or a vector according to the constrain. F1-score is also reported for individual landmarks and is computed as:

3 $$\begin{aligned} F1 = 2\times \frac{\mathrm{PPV} \times \mathrm{TPR}}{\mathrm{PPV}+\mathrm{TPR}} \end{aligned}$$

where $$\hbox {PPV}=\hbox {TP}/(\hbox {TP}+\hbox {FP})$$, $$\hbox {TPR}=\hbox {TP}/(\hbox {TP}+\hbox {FN})$$, $$\hbox {TP}=\hbox {diag}(\hbox {CM}(.))$$, $$\hbox {FP}=\hbox {sum}(\hbox {CM}(.),0)-\hbox {diag}(\hbox {CM}(.))$$, $$\hbox {FN}=\hbox {sum}(\hbox {CM}(.),1)-\hbox {diag}(\hbox {CM}(.))$$.

For the CNN model training, we set the batch size to 16 and the number of epochs to 100. We used the stochastic gradient descent (SGD) as the optimiser with a learning rate of 0.001 and momentum of 0.9. During fivefold cross-validation, model weights with the best accuracy on validation set are retained for the evaluation on the test fold. Our models are implemented in PyTorch and are trained using an Nvidia Titan Xp GPU and an Intel Xeon Silver 4114 CPU.

### Quantitative evaluation of the CNN models

For the purpose of choosing the CNN model with the best performance, ResNet-50 [7], Inception-v3 [19], VGG-11 [18] with batch normalisation (BN) [9], VGG-16 [18] with BN and DenseNet-121 [8] were tested on the four datasets (Table 2) that we organised based on our proposed guideline and British guideline [1, 16]. The measured overall accuracies are given in Table 3. We observe from this table that the CNN models trained without the NA class always perform significantly better than the models trained with the NA class. Datasets with the NA class add ambiguity during training as it contains images which may partially resemble other classes. The Inception-v3 and DenseNet-121 cost around 5 hours for training. The ResNet-50, VGG-11-bn and VGG-16-bn cost less time, which is around 3 hours, for the same training processes.

The average overall accuracy of these four models shows that DenseNet-121 gave slightly better accuracy followed by Inception-v3, VGG-16-bn, ResNet-50 and VGG-11-bn as shown in Table 3. Note that all CNN models performed equally good that demonstrate their strong learning capability and the practicality of our anatomical classification guideline. We choose DenseNet-121 as our backbone network structure for evaluating individual landmark classification in different guidelines because of its superior performance over other networks.

**Table 3:** Overall accuracy (%) of five CNN models for four datasets

No. (cite)	ResNet-50	Inception-v3	VGG-11-bn	VGG-16-bn	DenseNet-121
0 (proposed)	90.75	91.04	89.29	90.41	**91**.**11**
1 (proposed)	82.53	**82**.**56**	82.40	82.10	82.24
2 ([1, 16])	93.11	93.00	**94**.**00**	93.50	93.90
3 ([1, 16])	84.51	**85**.**26**	84.62	85.23	85.23
Means	87.72	87.97	87.43	87.81	**88**.**11**
STDs	4.34	4.22	4.25	4.43	4.62

The bolded values are the best overall accuracy rates under each of the data arrangements

**Table 4:** The F1-score (%) of DensetNet-121 on four datasets

GL	NA	PX	ES	SJ	FS	MA	MR	AS	LB	AM	DB	DD
0	–	**94.34**	**94.58**	**90.83**	93.54	91.90	**76.39**	89.40	**55.86**	92.76	**88.85**	**94.92**
1	68.28	79.25	88.35	82.92	90.03	84.12	74.50	80.82	52.71	87.98	80.31	93.76
2	–	–	94.02	88.42	**98.07**	**95.41**	–	**93.02**	–	**94.39**	88.63	94.22
3	**71.33**	–	89.78	83.30	92.16	87.32	–	85.84	–	88.84	80.76	93.24

GL, guideline. The bolded values are the best F1-score rates for each of the landmarks

**Fig. 5:**
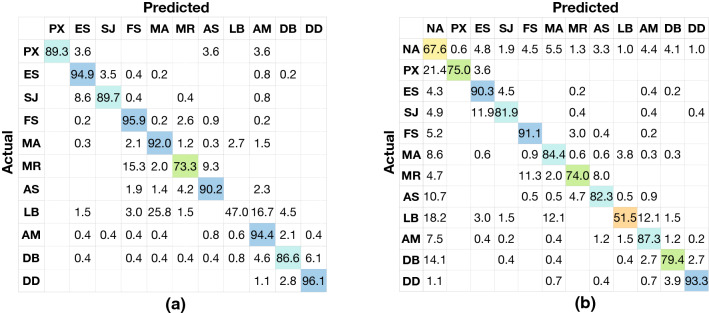
Confusion matrix for the model based on **a** the proposed guideline without NA and **b** the proposed guideline with NA. The actual labels are on the left side of the confusion matrix, and the predicted labels are on the bottom of the confusion matrix. **a** has 11 classes, and **b** has 12 classes. The values on the diagonal grids showed the recall rates (%) of each class, respectively, for the matrix. Referring to the colour of the colour bar and the corresponding number, the sample density in the confusion matrices is shown by colours

### Quantitative evaluation of the guideline

**Fig. 6:**
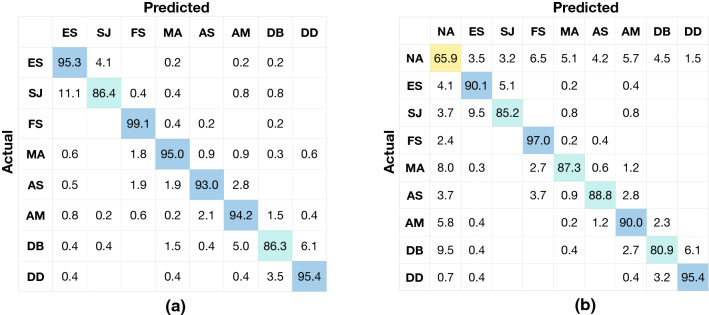
Confusion matrix for the model based on **a** the British guideline [1, 16] without NA and **b** the British guideline [1, 16] with NA. The actual labels are on the left side of the confusion matrix, and the predicted labels are on the bottom of the confusion matrix. **a** has eight classes and **b** has nine classes. The values on the diagonal grids showed the recall rates (%) of each class, respectively, for the matrix. Referring to the colour of the colour bar and the corresponding number, the sample density in the confusion matrices is shown by colours

Evaluation results for the proposed guideline and British guideline with/without the NA class are reported in Table 4 displaying the F1-score accuracy of individual classes, and their corresponding confusion matrices are shown in Figs. 5 and 6. The proposed guideline helps the CNN model to recognise three additional landmarks (PX, MR and LB) than the British guideline. The CNN model evaluated on our trimmed dataset corresponding to the British guideline (since NA, PX, MR and LB are excluded) achieved superior performance as shown in Fig. 6. The recall rates on the diagonal of the confusion matrix (Fig. 6) are 95.3%, 86.4%, 99.1%, 95.0%, 93.0%, 94.2%, 86.3% and 95.4% for ES (class 0), SJ (class 1), FS (class 2), MA (class 3), AS (class 4), AM (class 5), DB (class 6) and DD (class 7), respectively. With the addition of more landmarks (PX, MR and LB) as shown in Fig. 5a in the case of our proposed guideline, the performance of the CNN model on several individual landmarks remained almost the same as before, such as ES (class 1), SJ (class 2), AM (class 8), DB (class 9) and DD (class 10). The performance is low for LB (class 7) since it is hard to find a reference to well recognise LB from a single image.

From the confusion matrices in Figs. 5 and 6, we observe that the classification errors are mainly caused by three reasons:

- A small amount of landmarks are misclassified to NA by the CNN model. The CNN model without NA (number 0 and 2) outperformed their counterparts with NA (number 1 and 3) by 8.87% and 8.67% overall accuracies (Table 3) because several images are misclassified to NA. 21.4% PX (class 1), 5.2% FS (class 4), 8.6% MA (class 5), 10.7% AS (class 7), 18.2% LB (class 8), 7.5% AM (class 9) and 14.1% DB (class 10) are misclassified to NA (class 0) as shown in Fig. 5b. 8.0% MA (class 4), 5.8% AM (class 6) and 9.5% DB (class 7) were misclassified to NA (class 0) as shown in Fig. 6b.
- Several unqualified images (NA) were misclassified to landmarks by the CNN model. 32.4% and 34.1% NA were misclassified to landmarks by the method based on proposed guideline and by the method based on the British guideline, respectively. The images in NA are very different in appearance from each other. Artificial noise images, blurry images, pathological images and transitional images from all locations are all covered by this class. Hence, it is possible to have the feature representation of images in NA very similar to some of our labelled landmarks.
- Several landmarks with similar tissue appearances are easily misclassified to each other. Regardless of NA, anatomically adjacent landmarks are also easily confused by the CNN model. As illustrated in Fig. 5a, 8.6% SJ (class 2) was classified to ES (class 1); 15.3% and 9.3% MR (class 5) were classified to FS (class 3) and AS (class 6), respectively; 25.8% and 16.7% LB (class 7) were classified to MA (class 4) and AM (class 8), respectively; 6.1% DB (class 9) was classified to DD (class 10). As illustrated in Fig. 6a, 11.1% SJ (class 1) was classified to ES (class 0); 5.0% and 6.1% DB (class 6) were classified to AM (class 5) and DD (class 7), respectively.

We observe from our experimental results that CNN models fine-tuned for specific data distribution are useful for automatically identifying different anatomical sites. Our work shows the potential of using this method for quantifying photo-documentation. Furthermore, our proposed guideline showed the capability of recognising additional anatomical sites of interest compared to the British guideline. Capturing and recognising more anatomical sites are always the preferred choice of EGD clinicians as it helps in providing an elaborate analysis.

## Discussion and conclusion

In this paper, we proposed a deep learning-based anatomical site classification method for EGD images. Our work contained five consecutive steps, namely (a) data collection and preparation, (b) ROI extraction, (c) proposed guideline-based anatomical annotation, (d) training CNN models and (e) model evaluation. Our experimental results demonstrated the feasibility and effectiveness of our proposed guideline for training CNN models using only a small number of EGD images. The quantitative evaluation demonstrated that different CNN architectures performed equally good on our dataset. Moreover, the evaluation on individual images gave an insight into the robustness of different landmarks detection and the source of errors. We find that the proposed method has promising performance in discriminating unrepresentative landmarks (such as LB and MR) apart. More landmarks could provide a more elaborate analysis for precise diagnosis of EGD in the clinic.

Anatomical site classification for individual EGD images from the reports is a challenging problem since no temporal information is present. To further improve the results, we plan to analyse EGD videos in future using 3D CNN and recurrent neural networks, which will incorporate both spatial feature representation and temporal information simultaneously.
